# Mastoparan M extracted from *Vespa magnifica* alleviates neuronal death in global cerebral ischemia-reperfusion rat model

**DOI:** 10.22038/IJBMS.2022.60745.13461

**Published:** 2022-03

**Authors:** Mei Wang, Xiu-Mei Wu, Miao He, Heng Liu, Zhi-Bing Yang, Yue Li, Guang-Ming Wang, Hai-Rong Zhao, Cheng-Gui Zhang

**Affiliations:** 1 Yunnan Provincial Key Laboratory of Entomological Biopharmaceutical R&D, Dali University, Dali 671000, China; 2 National-Local Joint Engineering Research Center of Entomoceutics, Dali University, Dali 671000, China; 3 Genetic Testing Center, The First Affiliated Hospital of Dali University, Dali University, Dali 671000, Yunnan, China

**Keywords:** Brain ischemia, Hippocampal CA1 region, Neuroinflammatory diseases, Oxidative stress, Wasp venoms

## Abstract

**Objective(s):**

Global cerebral ischemia (GCI), a consequence of cardiac arrest (CA), can significantly damage the neurons located in the vulnerable hippocampus CA1 areas. Clinically, neurological injury after CA contributes to death in most patients. Mastoparan-M extracted from *Vespa magnifica* (Smith) can be used to treat major neurological disorders. Hence, this study aimed to assess the effects of Mastoparan-M on GCI.

**Materials and Methods:**

To evaluate the neurotoxicity and neuroprotective effect of Mastoparan-M, the CCK8 and Annexin V-FITC/PI apoptosis assays were first performed in hippocampal HT22 neuronal cells *in vitro*. Then, Pulsinelli’s 4-vascular occlusion model was constructed in rats. After treatment with Mastoparan-M (0.05, 0.1, and 0.2 mg/kg, IP) for 3 or 7 days, behavioral tests, H&E staining or Nissl staining, immunohistochemistry, and ELISA were employed to investigate neuroprotective effects of Mastoparan-M on GCI in rats.

**Results:**

*In vitro*, the growth of HT22 neuronal cells was restrained at concentrations of 30-300 µg/ml (at 24 hr, IC_50_=105.2 µg/ml; at 48 hr, IC_50_=46.81 µg/ml), and Mastoparan-M treatment (0.1,1 and 5 µg/ml) restrained apoptosis. *In vivo*, Mastoparan-M improved neurocognitive function and neuronal loss in the hippocampal CA1 area of rats. In addition, these effects were associated with the prevention of neuroinflammation, oxidative stress, and apoptosis.

**Conclusion:**

Mastoparan-M acts as a neuroprotective agent to alleviate neuronal death in rats.

## Introduction

Global cerebral ischemia-reperfusion injury (GCI/R) is commonly seen in emergency medicine. Restoration of spontaneous circulation (ROSC) after a cardiac arrest (CA) is the most common type of GCI/R in clinical practice. When encountering ROSC after CA, <9% of patients survive with good neurological outcomes (1) because the sudden cessation of blood flow drastically reduces glucose and oxygen supply to the brain, causing severe neuronal injury, including oxidative stress, metabolic dysfunction, and neuronal death (2, 3). The hippocampal region, particularly within the CA1 area, is most extensively involved in neuronal injury (4). This type of neuronal death in the hippocampal CA1 region begins 2–3 days after reperfusion and matures 7 days after the insult. This is known as apoptotic neuronal cell death (5, 6). Several biomechanisms are involved in the pathology of cerebral ischemia-reperfusion damage, including inflammation, oxidative stress, and apoptosis. Thus, it is critical to restore damaged neuronal structures and prevent neuronal loss for GCI/R treatment. The search for a cure remains a challenge in targeting neuronal apoptosis. 

Wasp venom extracted from *Vespa magnifica *(Smith) represents a complex mixture of biologically active proteins and peptides, such as phospholipases, hyaluronidase, phosphatase, α-glucosidase, serotonin, histamine, dopamine, noradrenaline, and adrenaline, with significant pharmacological effects and biological activity. Clinically, compounds extracted from wasp venom have been used as anti-inflammatory medicines to relieve pain and treat chronic inflammatory diseases, such as rheumatoid arthritis (7) and multiple sclerosis (8). Additionally, compounds extracted from wasp venom have been used to treat major neurological disorders, including epilepsy (8, 9), Parkinson’s disease (PD) (10-12), and Alzheimer’s disease (AD) (13). Mastoparan and bradykinin are exclusive to wasps (14-17). Recently, Mastoparans were classified as cell-penetrating peptides (18), while bradykinin was used to protect apoptosis-like delayed neuronal death in post-ischemic rat hippocampus (19, 20). Furthermore, increasing evidence has shown that bradykinin has significant anti-inflammatory properties and inhibits the activation of microglia by down-regulating tumor necrosis factor-alpha (TNF-α) and interleukin-1beta (IL-1β) (21). These findings suggest that wasp venom can be further explored for treatment in the pathology of GCI/R. 

Our previous studies have confirmed that wasp venom (0.125, 0.25, and 0.5 mg/kg) alleviated paw swelling and decreased arthritis scores in rheumatoid arthritis rats (22). Four compounds in the wasp venom have been purified and identified, including 5-Hydroxytryptamine, Vespakinin-M, Mastoparan M, and Vespid chemotactic peptide M (23). Interestingly, the analogs of Vespakinin-M and Mastoparan-M have neuroprotective effects (24-26). Mastoparan-M accounts for 70-80% of crude venom. However, no study has reported the effect of Mastoparan-M on GCI/R. Therefore, to evaluate the neurotoxicity and neuroprotective effects of Mastoparan M, the CCK8 and Annexin V-FITC/PI apoptosis assay were first performed in hippocampal HT22 neuronal cells in *vitro. *Then, we investigated the neurologic impairment and neuronal loss of hippocampal CA1 on GCI/R using Pulsinelli’s 4-vascular occlusion (4-VO) in rats by treating with Mastoparan-M (0.05, 0.1, and 0.2 mg/kg, IP). Meanwhile, we evaluated the release of pro/anti-inflammatory cytokines (e.g., Il-1β, TNF-α, IL-6, IL-8, and IL-10), oxidative stress, energy metabolism, and apoptosis in this model.

## Materials and Methods

***Animals***

Adult male Wistar rats weighing 280-300 g were obtained from the Liaoning Changsheng Biotechnology Co, Ltd, with an animal certificate no. of SCXK(Liao)2015-0001. The handling and surgery of the rats were approved by the Animal Care and Use Committee of Dali University, China (Animal Ethics no.: DLULAC2017-0117). All the rats were housed in a specific pathogen-free facility under 12-hr light/dark cycles in a temperature-controlled environment (22-25℃) with 40-70% humidity. The rats had free access to food and water.

***Global cerebral ischemia (GCI)***

Except for the sham group, all the rats were subjected to the 4-VO model, as previously described (27, 28). In brief, the rats were deeply anesthetized with 5.0% isoflurane and maintained by inhalation of 1.5% Isoflurane driven by 100% oxygen flow using the EZ-Anesthesia system (Euthanex Corp., Palmer, PA). The isolated bilateral vertebral arteries (VAs) were occluded using electrocautery (0.5 mm), and the wound was closed. Then, common carotid arteries (CCAs) were gently isolated and occluded using a surgical clamp. Next, GCI was induced by clamping both arteries with miniature artery clips, and the incision was primarily sutured. After 15 min of GCI, the clips were removed, and blood circulation was restored to the brain from CCAs. The incision was sutured using 4-0 Mersilk. The rats in the sham group underwent the same procedure without blocking bilateral VAs and CCAs. During the procedure, the rats’ systemic arterial blood pressure and electroencephalograph (EEG) signals were consistently monitored.

***Drug treatment***

Mastoparan-M was provided at the National-Local Joint Engineering Research Center of Entomoceutics. To control the quality of the wasp venom, a method based on high performance liquid chromatography (HPLC) was developed for chemical fingerprint analysis as previously described (Zhou *et al.* 2019). Edaravone (EDA), a free radical scavenger (Biomedical Engineering Center, Hebei Medical University, China, Number: H20090353), was utilized as a positive control. Three rats were raised in a cage and marked in groups. Male Wistar rats were randomly divided into six groups, including (a) the sham group (n=16), (b) the GCI group with vehicle (n=16), (c) the GCI group with EDA (6 mg/kg) treatment (n=16), and (d) the GCI group with Mastoparan-M (0.05, 0. 1, and 0.2 mg/kg, IP) treatment (n=41), respectively. After GCI/R at 0 h, the rats within the vehicle and drug-treated groups were treated with normal saline (NS) solution or Mastoparan-M (0.05, 0.1, and 0.2 mg/kg, IP), respectively. All the rats were treated after ischemia-reperfusion at 0, 22.5, 70.5, and 118.5 hr. At 72 hr, brains were collected from half of the animals in each group.

***Behavior test***

After GCI/R at 0, 24, 48, 72, 96, 120, and 144 hr, behavioral tests were performed as described in Tables 1 and 2 (29, 30) and continuously assessed for 7 days. In Table 1, the total score is 25 points. The stroke index is categorized into three grades, with 0−3 points representing mild injury, 4−10 points representing moderate injury, and >11 points (including death) representing severe damage. In Table 2, the total score is 10, with 0 representing normal, 1-3 representing mild damage, 4-6 representing moderate damage, and 7-10 representing severe damage.

***Hematoxylin-eosin (HE) and toluidine blue (Nissl) staining***

T**he r**ats were anesthetized on day 3 or day 7 after cerebral reperfusion (I/R). The brains were collected and fixed in 4% paraformaldehyde (PFA) for 12−18 hr. Then, the brain was dehydrated by an automatic dehydrator (Leica ASP-300S, Germany) and embedded in paraffin (Biological Tissue Embedding Machine, Xiaogan Hongye Medical Instrument Co, Ltd, model: BM-VIII). Then, the brain was coronally cut (4 µm) at the level of the hippocampus (bregma: -3.00 to -3.80 mm) using a rotatory microtome (Leica RM2245, Germany). The sections were then subsequently stained with H&E or Nissl staining. Sections were selected from each rat, and images of the hippocampus CA1 area were provided, respectively. The cells were counted and analyzed by ImageJ software. The average number of surviving cells on the right and left sides (neurons per 1 mm linear length in a single section of the dorsal hippocampus) was calculated for each rat. Three sections from each animal were used for counting. 

The calculation formula of neuronal density is: 

$\mathrm{neuron density}/\mathrm{mm}=\frac{\mathrm{Normal number of neurons in the hippocampus CA1 area}}{\mathrm{Totlal lenght in the hippocampus CA1 area}}\times 100$

***Biochemical estimations***

The rats were decapitated under anesthesia either on day 3 or day 7 post-GCI. Then, the hippocampus was collected, weighed, and homogenized. Using NS as the homogenization medium, 10% tissue homogenate was prepared and centrifuged at 3500 rpm for 10 min to acquire the supernatant, which was packed into the EP tube and kept at -80°C for future use. Lactic acid (LD kit, 20171014), lipid peroxide (LPO kit; 20170926), malondialdehyde (MDA kit; 20171011), nitric oxide synthase, (NOS kit; 20171014), nitric oxide (NO kit; China, 20171013), and superoxide dismutase (SOD kit; 20171012) were all purchased from Nanjingjiancheng Bioengineering Institute, in China, measured as previously described (31, 32), and employed according to manufacturers’ instructions. 

***Cytokine enzyme-linked immune sorbent assay (ELISA)***

The rats were decapitated under anesthesia on day 3 or day 7 after GCI. IL-1ꞵ (#147425023), TNF-α (#147881045), IL-6 (#146379036), and IL-10 (#152025018) were purchased from Thermo Fisher Scientific. According to manufacturer specifications, the levels of these cytokines were measured by ELISA kits.

***Immunohistochemistry***

Immunohistochemical staining (IHC) was conducted to identify the expression of cysteine aspartate specific proteinase-3 (Caspase-3), B-cell lymphoma-2 (Bcl-2), or c-fos in the hippocampus. Briefly, antigen retrieval was carried out by a 10-mM sodium citrate buffer at pH=6.0 in a microwave for 20 min at 100ºC. These sections were incubated for 1 h in the blocking solution (0.1% Triton-X, 10% normal goat serum in 1**×**PBS) at room temperature (RT). Then, the primary antibody Caspase-3 (1:200, Abcam, US, ab184787), Bcl-2 (1:200, Abcam, US, GR80570-12), c-fos (1:200, Abcam, US, GR3175387-1), and cleaved Caspase-3 (1:200, CST, #9664) were added to each section, respectively, and placed overnight at 4°C. The samples were then incubated using biotin-labeled secondary antibodies at RT for 30 min. The streptavidin-horseradish peroxidase (HRP) working medium was added and incubated at RT for 30 min. The positive signals of protein expression of Caspase-3, Bcl-2, and c-fos were localized to the cell cytoplasm, manifested as pale yellow, brown, or sepia positive granules. The positivity rate = (positive cells/total cells per field) ×100%.

***Cell cultures, treatment, and OGD***

HT22 hippocampal neuron cells were routinely cultured with a complete medium (DMEM containing 10% FBS, 100 U/ml penicillin G, and 100 mg/ml streptomycin) in a conventional incubator (37°C, 5% CO_2_, unlimited O_2_ content). The experiments were divided into eight treatment groups: the control group, the oxygen and glucose deprivation (OGD) group, and Mastoparan-M (0.1, 1, and 5 µg/ml). To establish the OGD/reperfusion (R) model, HT22 cells were incubated with DMEM free of glucose and FBS and placed in an anaerobic incubator defined as 1% O_2_, 5% CO_2_, and 94% N_2_ at 37 °C. After 18 hr of OGD, the cells were changed to a complete medium and reoxygenated in a conventional incubator for 8 hr. 

***CCK-8 assay***

Cell viability was measured by the CCK-8 assay. In detail, HT22 cells were seeded on 96-well plates and treated with different concentrations of Mastoparan-M (3, 10, 30, 100, and 300 µg/ml). Cells treated with DMSO (0.1%, v/v) served as the vehicle control. After 24 hr of incubation, the OGD/R model was established, and 10 μl of the CCK-8 reagent was added to each well. The plate was incubated at 37°C for 4 hr. Absorbance was surveyed at 450 nm by a Multiscan Spectrum (Spark, Tecan, Switzerland).

***Annexin V-FITC/propidium iodide assay kit***

After OGD/R, HT22 **cells **were treated with Mastoparan-M (0.1, 1, and 5 µg/ml) for 24 hr. The FITC Annexin V Apoptosis Detection Kit (556419) was purchased from BD Biosciences. HT22 (10^5^/well) cells were stained with this kit following the manufacturer’s instructions (http://www.bdbiosciences. com/ds/pm/tds/560931.pdf).

***Statistical analysis***

Statistical analyses were conducted using Graph Pad Prism 8 software. Kolmogorov-Smirnov test was used to determine the normal distribution of the samples. If the distribution of the sample was normal, we conducted statistical analyses in multiple groups using one-factor analysis of variance (ANOVA), followed by Dunnett’s test or Student’s t-test. If the samples were not distributed normally, a Kruskal-Wallis test was performed. A statistical difference was established when *P*<0.05. Chi-squared test was used to rank data, such as the behavioral test.

## Results

***Mastoparan-M extracted from wasp venom could be active components to restrain apoptosis in vitro***

**Figure 1:**
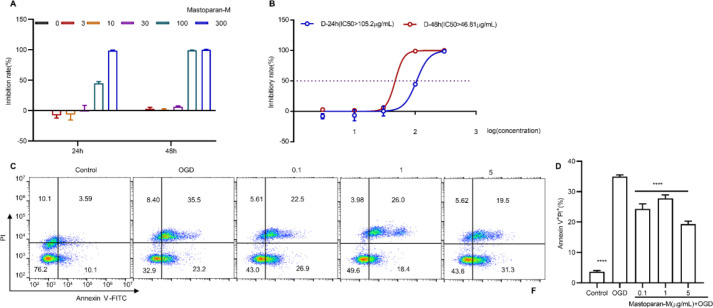
Mastoparan-M extracted from wasp venom could be an active component to inhibit apoptosis. (A-B) To evaluate the neurotoxicity of Mastoparan-M, a CCK8 assay was first performed in hippocampal HT22 neuronal cells. (C-D) Neuronal apoptosis was measured and quantified using an FITC Annexin V Apoptosis Detection Kit by flow cytometry. Data are mean ± SD (n=6) (****P<0.0001 vs. OGD/R)

To evaluate the neurotoxicity of Mastoparan-M, the CCK8 assay was performed in hippocampal HT22 neuronal cells. After treatment at 24 hr or 48 hr, the growth of HT22 neuronal cells was inhibited at concentrations of 30–300 µg/ml (at 24 hr, IC_50_=105.2 µg/ml and at 48 hr, IC_50_=46.81 µg/ml) (Figure 1A and B). Therefore, Mastoparan-M was used at concentrations of 0.1–5 µg/ml for all the cell experiments. Herein, we built an OGD/R model of HT22 hippocampal neuron cells, which could remarkably decrease cell activity and cause apoptosis in HT22 cells compared to the control group. However, Mastoparan-M treatment (0.1, 1, and 5 µg/ml) significantly inhibited apoptosis (Figure 1C and D). 

***Mastoparan-M relieved neurological deficit***

**Figure 2:**
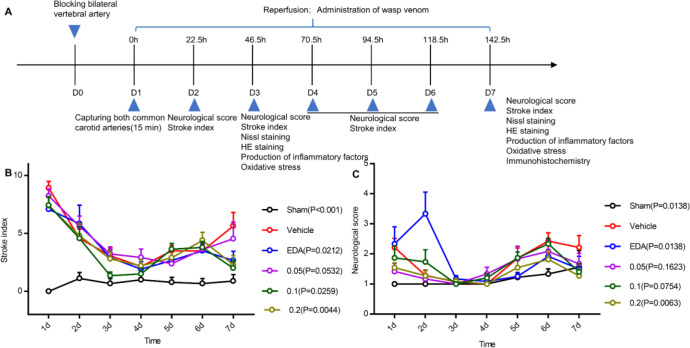
The effect of Mastoparan-M on neurological deficits after GCI/R. (A) The schedule of Mastoparan-M administration and behavioral assessment timeline is illustrated schematically. (B-C) Stroke index and neurology symptoms were evaluated according to the methods

During the process of GCI, various drug treatments and behavioral tests were conducted following the methods shown in Figure 2A. We evaluated the effect of Mastoparan-M treatment on animal behaviors previously mentioned (Tables 1 and 2). We assessed the severity of neurological deficit on day 7 using the stroke index and neurology symptoms (Table 3). All the rats survived in the sham group, and there were no significant differences in mortality rate and degree of disability between the vehicle and Mastoparan-M groups (Table 3). We determined changes in spontaneous motor activity for 7 days; these changes gradually disappeared. On day 7, the rats in the vehicle group demonstrated the worst symptoms of central nervous system (CNS) damage, such as areflexia, spastic paralysis of limbs, tonic tension of torso muscles, lateral position by stroke index, and neurology symptoms, compared to the sham group (*P*<0.01). In contrast, Mastoparan-M (0.2 mg/kg, *P*<0.05) decreased the stroke index (Figure 2B). Compared to the sham group, neurology symptoms in the vehicle group were remarkably increased (*P*<0.05) (Figure 2C). Additionally, treatment with Mastoparan-M (0.1 and 0.2 mg/kg) or EDA prevented GCI-induced neurological symptoms (Figure 2C, Table 3).

***Mastoparan-M prevented GCI-induced delayed neuronal cell death in the hippocampal CA1 region***

**Figure 3:**
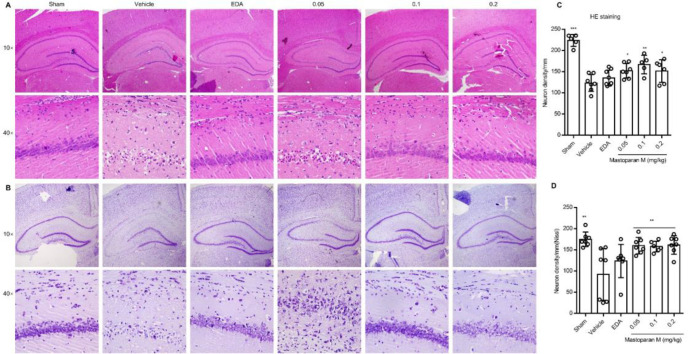
The effects of Mastoparan-M on the survival of neurons on day 3 after GCI/R. (A-B) Representative images of HE or Nissl staining in the hippocampus. (C-D) Quantification of the number of surviving neurons by HE and Nissl staining. Scale bars: 50 µm; magnification: ×4 or ×40. Values are expressed as mean ± SD, n=6-8 for each group, *P<0.05, **P<0.01 vs. the vehicle group

**Figure 4:**
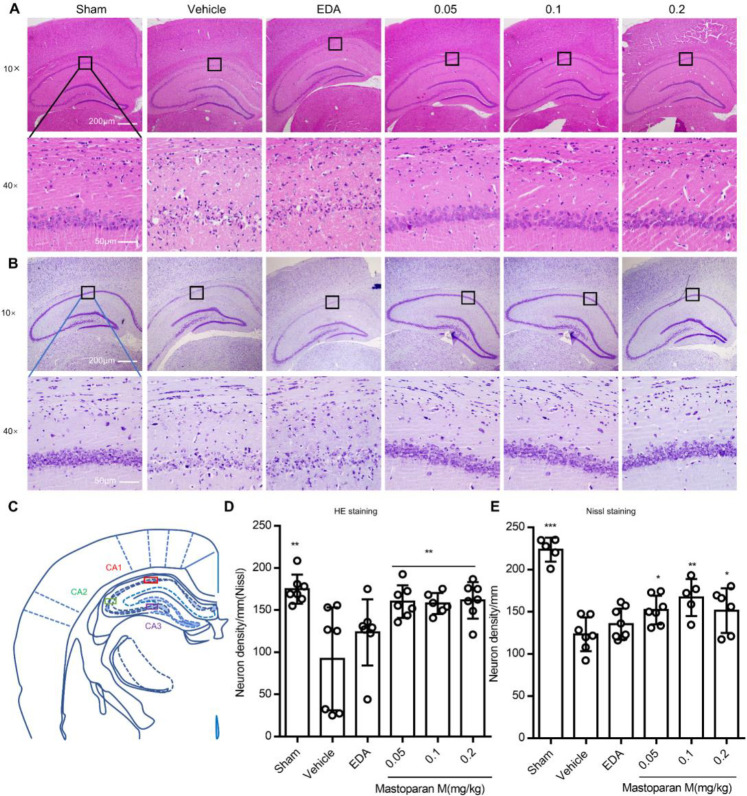
The effect of Mastoparan-M on the survival of neurons on day 7 after GCI. (A) Representative images of H&E staining in the hippocampus. (B) Representative images of Nissl staining in the hippocampus. (C) The hippocampal CA1 region was described in detail. (D-E) Quantification of the number of surviving neurons by H&E and Nissl staining. Scale bars represent 50 µm; magnification is ×4 or ×40. Values are expressed as mean ± SD; n=8–11 for each group; *P<0.05 or **P<0.01 vs. the vehicle group

Next, we evaluated whether treatment with Mastoparan-M could protect the hippocampal CA1 region against GCI-induced delayed neuronal cell death on day 3 or day 7. The drug administration schedule and behavioral assessment timeline are schematically illustrated in Figure 2A. The neurons of the sham group retained their structural and functional integrity, and the labeled neurons were organized closely (Figure 3A-B). Compared with the sham group, massive neuronal death in the hippocampal CA1 region led to neuronal depletion of layers and disorganization of the remaining neurons in vehicle-treatment rats by H&E and Nissl staining on day 3 (Figure 3A-B) and day 7 (Figure 4A-B). Furthermore, the hippocampal CA1 region and counts of the observed neurons were described in detail (Figure 4C). The quantitative morphological analysis demonstrated a significant decrease in the proportion of neurons in the vehicle group compared to the sham group (*P*<0.01). In addition, administration of Mastoparan-M decreased the severity of the pathomorphological changes in the hippocampal CA1 region on day 3 (Figure 3C-D) and on day 7 (Figure 4D-E). 

***Mastoparan-M blocked oxidative stress and restored energy metabolism in the hippocampal CA1 region***

**Figure 5:**
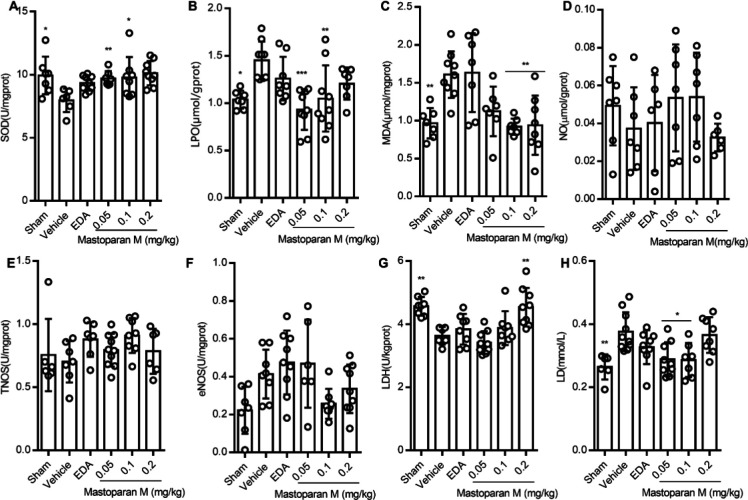
Mastoparan-M reduces oxidative stress after GCI/R in rats. (A) The effect of Mastoparan-M on SOD activity. (B-C) Lipidperoxidation after GCI/R was demonstrated by levels of LPO and MDA in the hippocampal CA1 region. (D-F) NO, NOS, and eNOS activity were detected after GCI/R. (G-H) ATP synthase and LD were determined to examine the effect of Mastoparan-M treatment on energy metabolism after reperfusion injury. Data is represented by mean ± SD; n=7–10; *P<0.05 and **P<0.01

In order to assess whether Mastoparan-M treatment could diminish oxidative stress induced by GCI/R in rats, we evaluated the markers of lipid peroxidation, including SOD, MDA, and LPO. SOD activity was found to be increased in vehicle-treated rats after GCI/R, in comparison to the sham-operated animals, and was further increased by Mastoparan-M treatment (0.05 mg/kg, *P*<0.01; 0.1 mg/kg, *P*<0.05) (Figure 5A). Accordingly, high levels of LPO and MDA were observed in vehicle-treated rats after GCI/R compared to the sham group (LPO, *P*<0.01; MDA, *P*<0.05) (Figure 5B-C). Mastoparan-M significantly decreased LPO levels (0.1 and 0.2 mg/kg, *P*<0.01) and MDA (0.05 mg/kg, *P*<0.001; 0.1 mg/kg, *P*<0.01), compared to the vehicle group (Figure 5B-C).

NO, total nitric oxide synthase (TNOS), and endothelial nitric oxide synthase (eNOS) activities in the brain of the rats in the vehicle group were not significantly higher compared to the sham group (Figure 5D-F). Compared to the vehicle group, the Mastoparan-M group might not be implicated in the prevention of endothelial dysfunction after GCI/R. We found that the Mastoparan-M group (0.2 mg/kg, *P*<0.5) exhibited increased LDH content compared to the vehicle group (Figure 5G). In addition, there was an evident decrease in LD levels in the Mastoparan-M group (0.05, 0.1 mg/kg, *P*<0.05) (Figure 5H) compared to the vehicle group.

***Mastoparan-M inhibited the release of pro-inflammatory cytokines in the hippocampal CA1 region***

**Figure 6.**
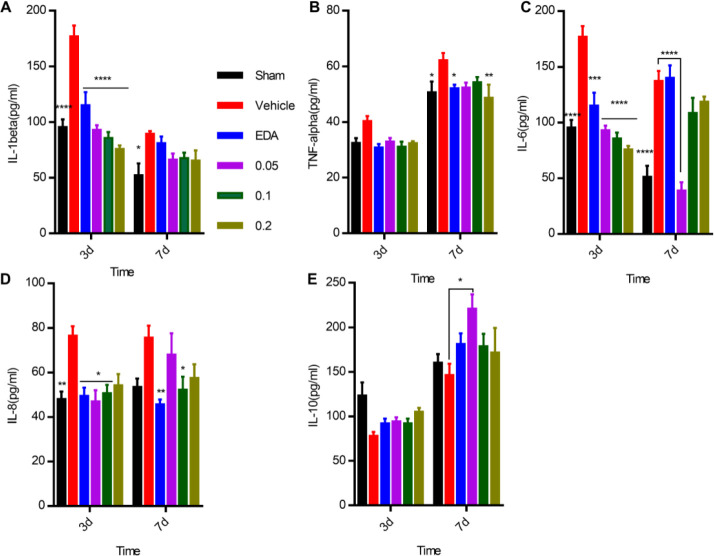
Mastoparan-M regulated inflammatory mediators, including the release of pro-inflammatory and anti-inflammatory mediators on day 3 or day 7 after GCI. (A-D) Mastoparan-M reduced the release of pro-inflammatory mediators, including TNF-α, IL-1β, IL-6, and IL-8. (E) Wasp venom increased the release of anti-inflammatory IL-10. The results are expressed as mean ± SD; n = 6–13; *P<0.05, **P<0.01 vs. the vehicle group

The IL-1β concentration of the hippocampal CA1 region was found to significantly increase on day 3 or day 7 after GCI/R (*P*<0.001 and 0.05, respectively; Figure 6A), and decrease significantly by Mastoparan-M (0.05, 0.1, and 0.2 mg/kg) or EDA treatment at day three (*P*<0.0001) (Figure 6A). TNF-α concentrations were elevated on day 3 or day 7 after GCI/R in the vehicle group, decreasing significantly by Mastoparan-M treatment on day 7 (0.2 mg/kg, *P*<0.01), but not on day 3 (Figure 6B). Treatment with Mastoparan-M on day 3 or day 7 led to a significant decrease in IL-6 levels compared to the vehicle animals. In particular, IL-6 levels were significantly lower in the Mastoparan M-treated group (0.05, 0.1, and 0.2 mg/kg) on day 3 compared to the Mastoparan M-treated group (0.1 and 0.2 mg/kg) on day 7 after GCI/R (Figure 6C). Compared with the sham group, IL-8 levels significantly increased on day 3 (*P*<0.01) and day 7 (*P*<0.05) after GCI/R, decreasing after treatment with Mastoparan-M on day 3 (0.05 and 0.1 mg/kg, *P*<0.05) and day 7 (0.1 mg/kg, *P*<0.05), respectively (Figure 6D). Compared with the sham group, IL-10 levels significantly decreased in the vehicle-treated rats on day 3 or day 7. In contrast, Mastoparan-M (0.05 mg/kg) increased the release of IL-10 (*P*<0.05, Figure 6E). 

***Mastoparan-M suppressed the apoptotic pathway in the hippocampal CA1 region following GCI/R***

**Figure 7:**
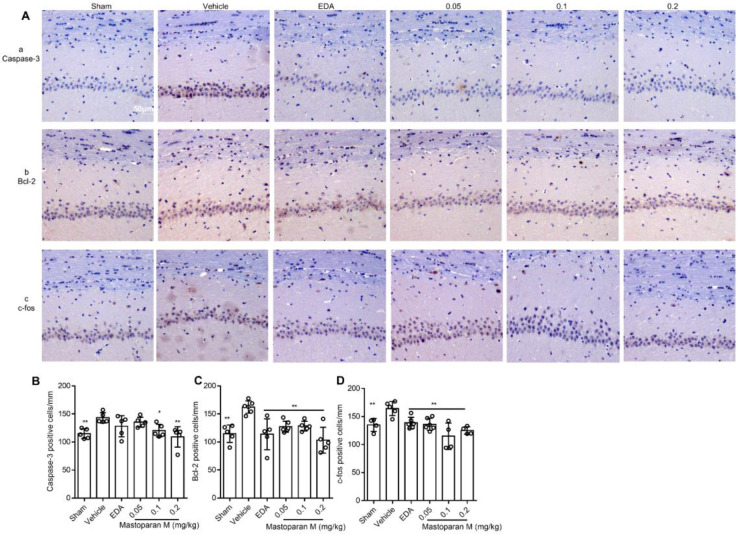
Changes in the expression of apoptosis-related proteins in the hippocampus of GCI rats. (A) Representative immunohistochemistry (IHC) for Caspase-3, Bcl-2, and c-fos expression in hippocampal CA1 region by Mastoparan-M treatment after GCI. (B-D) The number of Caspase-3-, Bcl-2-, and c-fos-positive cells in the hippocampal CA1 were normalized and given as cells/mm. Three micrographs with different magnifications are shown for experimental groups. Scale bar represents 50 μm; n = 5–6 per group. Data are presented as mean ± SD; *P<0.05; **P<0.01 vs. the vehicle group

GCI/R evoked a significant increase in the activities of caspase-3, Bcl-2, and c-fos, compared to the sham group (Figure 7A and 7B-D). Importantly, compared to the vehicle-treated rats, increased activities were dramatically inhibited in the Mastoparan-M-treated rats, including caspase-3 (0.2 and 0.6 mg/kg, *P*<0.05), Bcl-2, and c-fos (0.05, 0.1, and 0.2 mg/kg, *P*<0.01) (Figure 7B-D). 

**Table 1:** The calculation of combined behavioral score for stroke index

**Symptom**	Score
Hair was dirty and shaking	1
Reduced or retarded movement	1
Ototactile retardation	3
Raise their head	3
Hind limb outspread shows character “8”	3
Severe ptosis	1
Turn around	3
Convulsions or explosive movements	3
Extremely frail	6

**Table 2:** Neurological symptom score

**Symptom**	Score
There was the spontaneous exploration	0
They can walk when stimulated	1
Normal gait	0
Ataxic gait	1
Awkward gait (crawl)	2
No gait	3
Can eat food	0
Not eating	1
They can drink water spontaneously	0
Unable to drink water spontaneously	1
The pain stimulated the rat, and it moved	0
The pain stimulated the rats, which moved only the head or trunk	1
The pain stimulated the rats, which retracted or did not respond	2

**Table 3:** The effect of mastoparan M on neurological deficit

Groups	Dose (mg/kg)	Functional neurologic impairment			P-value is Fisher’s exact probability (χ^2^ test)
		Mild	Moderate	Severe	vs. the vehicle group
Sham	-	11	5	0	0.035
Vehicle	-	6	7	3	-
EDA	6	9	5	2	0.567
Mastoparn-M	0.05	8	3	3	0.415
	0.2	7	3	3	0.415
	0.6	9	6	0	0.085

Using the contingency table chi-squared test, the overall χ2 = 9.830 a. The total *P*-value = 0.456

## Discussion

Previous studies have demonstrated that bee venom treatment, which normalized some of the neuro-inflammatory and apoptotic markers and restored brain neurochemistry, is a promising neuroprotective therapy for PD (10, 33-35). However, compared to bee venom, there is a lack of pharmacological investigations for the use of wasp venom as a treatment. Our previous data showed that the main components of wasp venom are 5-hydroxytryptamine, vespakinin-M, Mastoparan-M, and vespid chemotactic peptide M, respectively. Recent research on the peptide has focused on the cell-permeable peptide, particularly Mastoparans (18), which serve as vehicles for delivering different molecules and particles into the brain and neurons, and have been studied in combination with compounds that act on the CNS (36). Increasing evidence has shown that vespakinin-M has effective anti-inflammatory properties and inhibits the activation of microglia by down-regulating TNF-α and IL-1β (21). Thus, over the last decade, natural, modified, or chimeric Mastoparans or vespakinin-M have been utilized as a potential treatment for several neurological conditions (37). Mastoparan-M is an amphipathic tetradecapeptide (Ile-Asn-Leu-Lys-Ala-Ile-Ala-Ala-Leu-Ala-Lys-Lys-Leu-Leu-NH2) toxin and a vespid venom Mastoparan counterpart isolated from *Vespa magnifica* (Smith) in China.

Herein, we firstly confirmed that Mastoparan-M extracted from wasp venom could have active components to inhibit apoptosis *in vivo *and *in vitro*. GCI/R leads to delayed neuronal damage in certain vulnerable regions of the brain, such as the hippocampal CA1 region, in both patients and experimental animals (38, 39). After an ischemic insult, transient mitochondrial swelling with the disintegration of cristae, cytoplasmic vacuolation, disaggregation of polyribosomes, a reduction in the rough endoplasmic reticulum, and loss of Golgi apparatus cisterns and vesicles were immediately observed within the CA1 neurons (40, 41). Then, in approximately 5*-*10 min, there was a massive proliferation of membranous cytoplasmic organelles, followed by an overt cellular disintegration, as seen in the CA1 neurons by the end of day 4 (42). EDA is a new potent free radical scavenger that is clinically utilized to reduce neuronal damage in ischemic stroke. We reported that Mastoparan-M, in doses of 0.05, 0.1, or 0.2 mg/kg, reduces neurological deficits after GCI/R and attenuates neuronal damage in the hippocampus with reperfusion on day 3 and day 7. Our results are consistent with numerous studies showing the neuroprotective effects of bee venom or wasp venom and its positive impact on functional outcomes after ischemia in rodents (43). Bee venom (2-5 mg/kg) could exert anti-inflammatory and antinociceptive effects on the inflammatory reactions for multiple sclerosis in rats (44). Melittin (0.1 mg/kg, twice a week) could treat amyotrophic lateral sclerosis (45). We selected the dose (0.05, 0.1, or 0.2 mg/kg) and frequency (twice a week) of Mastoparan-M administration according to the neuroprotective effect of melittin in rats. 

Inflammation is an important contributor to the pathophysiology of cerebral I/R injury and exacerbates neuronal damage (46, 47). The most important cytokines related to inflammation in cerebral ischemia include IL-1β, TNF-α, IL-6, IL-10, and transforming growth factor-β (TGF-β) (48). Subsequently, we determined the release of inflammatory mediators in rats after GCI/R by Mastoparan-M treatment. Additionally, Mastoparan-M reduced the release of pro-inflammatory mediators, including TNF-α, IL-1β, IL-6, and IL-8 on day three or day seven after GCI. In addition, IL-10 levels increased in Mastoparan-M-treated rats after GCI.

Neuronal oxygen stores are depleted after the cessation of cerebral circulation, which is followed by anaerobic glycolysis, leading to the depletion of ATP and brain glucose (49). Herein, we showed that Mastoparan-M increased LDH levels and decreased (50) LD content compared to the vehicle group after GCI/R. Neuroinflammation is characterized by releasing cytotoxic factors, including reactive oxygen species (ROS), cytokines, nitric oxide, and matrix metalloproteinases. Although reperfusion restores cerebral blood flow, it can cause secondary brain injury by increasing ROS levels, which causes damage through lipid peroxidation, protein oxidation, and DNA fragmentation, all contributing to neuronal death (51). However, Mastoparan-M was associated with a significant decrease in LPO and MDA content in the hippocampal CA1 region, reflecting the comparability of oxidative stress therapy. Simultaneously, SOD activity increased in the Mastoparan-M-treated group. SOD has antioxidant activity that reduces ROS, protecting cells against oxidative stress. Interestingly, endothelial NOS (eNOS) inhibition increases infarct volume and reduces IkB-α expression within the ischemic brain (52, 53). Therefore, we determined NO, TNOS, and eNOS activities in the hippocampal CA1 region. However, there were no significant differences between the groups.

When cerebral ischemia occurs, endogenous repair mechanisms, including angiogenesis, nerve regeneration, and synaptic remodeling in the brain, are immediately initiated (54, 55). Bcl-2, a trigger of ischemic neuronal death, activates caspase-3 using the mitochondrial pathway. In this line, we demonstrated that Mastoparan-M is related to the neuronal apoptosis pathway in the hippocampus, following GCI/R. Therefore, the effects of Mastoparans on neuronal loss in this rat model were studied.

***Limitations of the study***

Firstly, we did not perform a learning/memory skills test (a Morris water maze, a “Y” maze or other similar tests) to evaluate spatial learning and memory disorders. Secondly, EDA, a free-radical scavenger and a neuroprotective agent, was approved in 2001 in Japan and China to treat patients with acute cerebral ischemic stroke. In clinical studies, edaravone improved the core neurologic deficits, activities of daily life, and functional outcomes of stroke patients [51, 52]. Thus, we selected EDA as a positive drug in this study. Regarding the anti-inflammatory effect, the selection of positive medications is not ideal. Finally, although Mastoparan-M exhibited a neuroprotective effect in this study and other studies (24), systemic toxicity tests for Mastoparan-M, such as liver/ kidney, and especially those related to coagulation, platelet function, and bleeding should be performed in the future.

## Conclusion

We demonstrated that Mastoparan-M attenuates neuronal loss in the hippocampal CA1 area after GCI/R in rats. These are associated with the prevention of neuroinflammation and oxidative stress and inhibition of the activation of the final apoptotic executioner, Caspase-3, Bcl-2, and c-fos. 

## Authors’ Contributions

ZCG and ZHR Study conception and design; WM and WXM Data analyzing and draft manuscript preparation; HE and WM Methodology; WXM Formal analysis; ZHR and ZCG Funding acquisition; YZB and ZCG Investigation; ZCG Project administration; LH Resources; ZHR Validation; LY Visualization; ZHR and WGM Roles/Writing - original draft; ZHR and ZCG Writing - review & editing.

## Data Availability

The data used to support the findings of this study are included within the article and can be made freely available

## Conflicts of Interest

The authors declare no conflicts of interest regarding the publication of this paper.
